# The Impact of Portal Satisfaction on Portal Use and Health-Seeking Behavior: Structural Equation Analysis

**DOI:** 10.2196/16260

**Published:** 2020-03-27

**Authors:** Reginald A Silver, Chandrasekar Subramaniam, Antonis Stylianou

**Affiliations:** 1 University of North Carolina at Charlotte Charlotte, NC United States

**Keywords:** patient portals, health care IT, technology usage, health-seeking behavior

## Abstract

**Background:**

Our study addresses a gap in the modern information systems (IS) use literature by investigating factors that explain patient portal satisfaction (SWP) and perceptions about health-seeking behavior (HSB). A novel feature of our study is the incorporation of actual portal use data rather than the perceptions of use intention, which prevails in the modern IS literature.

**Objective:**

This study aimed to empirically validate factors that influence SWP as an influencing agent on portal use and HSB. Our population segment was comprised of college students with active patient portal accounts.

**Methods:**

Using web-based survey data from a population of portal users (n=1142) in a university health center, we proposed a theoretical model that adapts constructs from the Technology Acceptance Model by Davis, the revised Technology Adoption Model by Venkatesh, the Unified Theory of the Acceptance and Use of Technology 2, and the Health Belief Model by Rosenstock et al. We validated our model using structural equation modeling techniques.

**Results:**

Our model explained nearly 65% of the variance in SWP (R^2^=0.6499), nearly 33% of the variance in portal use (R^2^=0.3250), and 29% of the variance in HSB (R^2^=0.2900). Statistically significant antecedents of SWP included social influence (beta=.160, t_499_=6.145), habit (beta=.114, t_499_=4.89), facilitating conditions (beta=.062, t_499_=2.401), effort expectancy (beta=.311, t_499_=11.149), and performance expectancy (beta=.359, t_499_=11.588). SWP influenced HSB (beta=.505, t_499_=19.705) and portal use (beta=.050, t_499_=2.031). We did not find a statistically significant association between portal use and HSB (beta=.015, t_499_=0.513). Perceived severity significantly influenced HSB (beta=.129, t_499_=4.675) but not portal use (beta=.012, t_499_=.488).

**Conclusions:**

Understanding the importance of SWP and the role it plays in influencing HSB may point to future technology design considerations for information technology developers and health care providers. We extend current Expectancy Confirmation Theory research by finding a positive association between SWP and portal use.

## Introduction

### Background

Patient portals have become instrumental in the engagement of patients in their own care. A number of factors have contributed to the proliferation and evolution of portal systems. Patient portals evolved out of the expansion of electronic health record (EHR) systems that were incentivized by the American Recovery and Reinvestment Act. The act required health care providers to implement EHRs as a mechanism to ensure patient safety, improve the coordination of patient care, and better engage patients and their caregivers, while maintaining privacy and security of personal health care information. Health care providers were instructed to ensure that at least 5% of their patients could access their health information over the Web and to ensure that they could also exchange secure messages with their health care provider [1]. Patient portals became an important tool in promoting the kind of patient engagement prescribed by the American Recovery and Reinvestment Act. Patient portals promote this engagement through functionality that includes the ability to exchange secure communication with health care providers, schedule appointments, review lab results, and renew prescriptions for medications. Even with all of these factors that would seemingly promote high levels of patient portal usage, patient portal usage has been relatively low since the inception of these systems. Peacock et al [2] estimate patient portal usage to be only about 28% for portal users within the first year of activating a portal account. Current literature has provided a basic understanding of factors that influence portal use and how this use is thought to influence outcomes. With this research study, we validate and extend this knowledge base.

Our research effort is intended to fill a gap in current health care information technology (IT) research concerning portal user perceptions about portals and how those perceptions are related to perceived health-seeking behavior (HSB) and satisfaction with the portal. We investigate how portal satisfaction (SWP) may be associated with perceived HSB and with portal use. Furthermore, our study adds to the literature by using objective portal use data as opposed to intention to use, which is prevalent in the current literature. Specific research questions (RQs) include the following:

- *RQ1*: What factors are associated with a patient’s portal satisfaction?
- *RQ2*: Does patient portal satisfaction influence health-seeking behavior?
- *RQ3*: Does patient portal use influence health-seeking behavior?
- *RQ4*: Does patient portal satisfaction influence portal use?
- *RQ5*: Does perceived severity of health condition influence health-seeking behavior?
- *RQ6*: Does perceived severity of health condition influence portal use?

We next review the current literature on patient portals to identify broad findings about and research gaps in portal use. Following this, we describe our research model and hypotheses. We then present our research methods and results. We conclude our paper with a discussion of our findings, limitations, and conclusions.

### Literature Review

Existing research has shown that patient portal usage varies by demographic factors such as age, sex, and morbidity level. Portal usage has been demonstrated to be higher among middle-aged female patients with more severe conditions [3]. Repeated portal usage has been demonstrated to be higher among patients with chronic medical conditions [4]. A patient is more likely to have an interest in using the portal to communicate with their health care provider when the patient is dissatisfied with the health care provider’s responsiveness to traditional forms of communication [5]. Other factors that have been demonstrated to influence portal usage include the endorsement of the portal by the health care provider, the utility of portal features, the usability of portal features, and the health literacy of the patient [6]. Conversely, portal usage has been hindered by low health literacy among end users [7].

Knowledge about patient portal usage comes primarily from studies that evaluate usage in the outpatient setting. In the inpatient setting, patient portal usage seems likely to have the same positive effects regarding self-involvement in one’s own care. Inpatient usage, however, has also been low at approximately 23% [8]. Similar patterns of usage can be seen across both the inpatient and outpatient settings. Patients with more severe conditions (eg, surgical patients), as demonstrated by longer lengths of stay, tend to use the portal more than patients with less severe conditions [9]. Portal usage across both inpatient and outpatient settings is impacted by demographic factors such as age, sex, education level, and race. However, a missing link is understanding how patient satisfaction impacts portal use.

Existing studies have been focused largely on describing portal usage through the analysis of system data, but understanding the factors that influence HSB still represents a gap in the existing literature [10]. Even fewer studies have been grounded in some theory-driven framework. Marton and Choo [11] identified only four such theory-driven studies in their 2011 paper on theoretical models for Web-based health information seeking. The theoretical frameworks utilized by those studies comprised expectancy value models, the Theory of Planned Behavior, the Technology Acceptance Model (TAM), and a behavioral model of Web-based information seeking. This gap in contemporary research on patient portal usage may be filled by the expanded use of theoretical frameworks such as the Health Belief Model (HBM), TAM, and the Unified Theory of the Acceptance and Use of Technology 2 (UTAUT2) [12-15].

Research on patient portal usage predicated upon theoretical frameworks is emerging. Tavares and Oliveira [16] used a derivative of Venkatesh’s UTAUT2 theoretical framework to investigate EHR adoption with an added construct of the patient’s self-perception. Unique to their study, Tavares and Oliveira integrated into their theoretical model the construct of patient self-perception that measured perceived vs real severity of a portal user’s chief medical complaint. Tavares and Oliveira found that statistically significant motivators for the behavioral intention to use a patient portal were performance expectancy, effort expectancy, habit, and self-perception. Similar to Tavares and Oliveira, we extend Venkatesh’s UTAUT2 model to include constructs and relationships for perceived HSB and perceived satisfaction with the portal.

Another stream of literature that has emerged focuses on understanding the relationship between user satisfaction with technology (eg, portals) and the actual use of the technology. Deng et al [17] studied the use of mobile internet services and found that user satisfaction positively impacted the intention to continue using the services. Ghobakhloo et al [18] reviewed different theoretical models of technology acceptance and formulated an integrated model of acceptance and satisfaction. Their model proposes that technology satisfaction impacts technology usage, which, in turn, impacts usage behavior [18].

With this research effort, we hope to inform the research community about portal user perceptions and how these perceptions are related to perceived HSB and satisfaction with the portal. We investigate how SWP may be associated with perceived HSB.

### Research Model and Hypotheses

**Figure 1:**
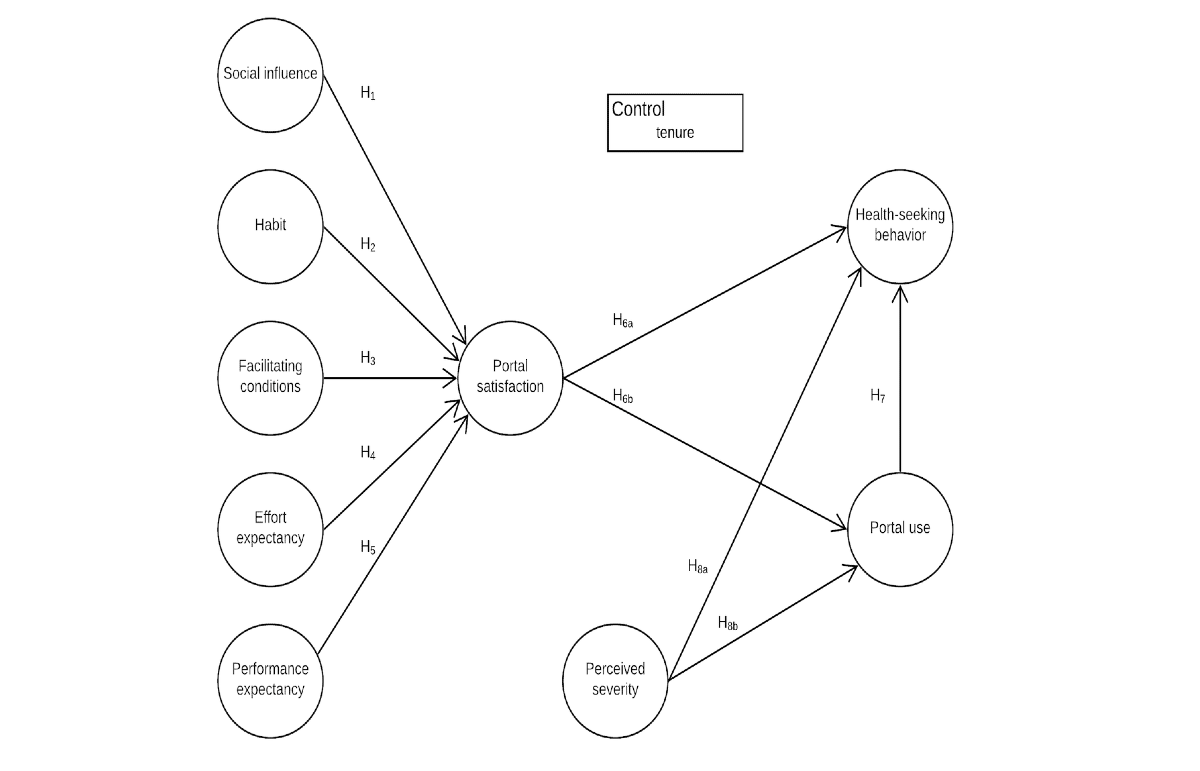
Proposed research model.

Our theoretical model incorporates social influence, habit, facilitating conditions, effort expectancy, and performance expectancy. Each of these constructs are traditionally used exogenous variables in management information systems (IS) research. We explore how these variables affect satisfaction with the portal and use. Drawing from HBM, we propose a relationship between perceived severity and HSB. Similarly, we propose a relationship between perceived severity and use. Our final hypothesis tests the relationship between portal use and HSB. Our complete research model is shown in Figure 1.

Patients who interact with health care technology, such as patient portals, are usually motivated to use the technology because it might be the main source of contact with their health care provider. Studies that have explored TAMs have consistently identified social influence as an important factor that influences end user behavior toward technology. Social influence is based on social contagion theory, which postulates that an individual’s behaviors are subject to those of the people who are important to that individual [19]. These influencers include care givers, health care providers, and close relatives. An example of social influence is when family members discuss the benefits of portals with the patient or are themselves users of portals [20].

Bhattacherjee [21] identified confirmation as an antecedent to technology satisfaction while proposing the use of the Expectation-Confirmation Model in an IS context. Bhattacherjee defined confirmation as the comparison between end users’ pre- and post-technology usage expectations. Similar to how Bhattacherjee positioned confirmation as an antecedent of technology satisfaction, we posit that it is feasible that a portal user’s social circle can serve as a proxy for confirmation expectation. Portal users with more positive input from their close social circle about portals are likely to be more satisfied themselves with the portal. Although there is little empirical research in this space, we test this influence on satisfaction in our model.

#### H1: Perceived Social Influence is Positively Associated With Portal Satisfaction

Venkatesh et al [22] considered the effect that habit has on technology use. Habit has been demonstrated to predict end user intention to use a technology. Contemporary views of habit have also relied on the definition provided by Limayem et al [23] who defined habit as the extent to which people tend to automatically execute certain behaviors such as technology use because of learning. Amoroso and Lim [24] found a positive correlation between habit and consumer satisfaction. As Amoroso and Lim did find a correlation between habit and satisfaction, we found utility in testing this correlation between habit and satisfaction in our theoretical model. Drawing from these previous uses of habit in IS research, we hypothesize that habit is positively associated with SWP.

#### H2: Habit is Positively Associated With Portal Satisfaction

Facilitating conditions is a UTAUT construct that describes a person’s perception of the resources and any other supporting elements at their disposal that assist them in performing a task (eg, technology use) [22]. Maillet et al [25] tested and found a statistically significant link between facilitating conditions and nurse satisfaction with an EHR. Conditions facilitating the access to and use of the system lead to a positive user experience, resulting in higher user satisfaction. Hence, we posit that facilitating conditions are positively associated with SWP.

#### H3: Facilitating Conditions are Positively Associated With Portal Satisfaction

The technological characteristics of the patient portal are important in motivating a patient to use the portal. Two important elements of portal technology are effort expectancy and performance expectancy. Effort expectancy is the degree to which the portal is easy to navigate and use, thus requiring less effort by the user [26]. An end user who finds a system intuitive and easy to use will have less frustration in completing tasks while using that system. In addition to finding the previously cited link between facilitating conditions and EHR satisfaction among nurses, Maillet et al [25] tested and found a statistically significant link between effort expectancy and satisfaction. Building on this finding by Maillet et al [25], we explore whether effort expectancy is associated with SWP.

#### H4: Effort Expectancy is Positively Associated With Portal Satisfaction

Performance expectancy is the degree to which the user perceives that the technology will help in carrying out functions important to the user [22]. For a patient portal user, performance expectancy reflects the patient’s perception of how well it will help with better management of the user’s health care. Some of the expected benefits of using a portal are viewing lab results on a smart device, easily scheduling or rescheduling appointments, and securely messaging physicians or nurses [27]. If these postadoption expectations are met, then portal users are likely to be satisfied with their portal. We find support for this idea through existing IS research that has used the Expectation-Confirmation Theory (ECT) to demonstrate a positive and statistically significant link between confirmation and satisfaction [28,29]. As technology users confirm that their expectations for the technology are being met, they exhibit satisfaction with that technology. We hypothesize that performance expectancy is positively associated with SWP.

#### H5: Performance Expectancy is Positively Associated With Portal Satisfaction

Evidence is accumulating in the medical literature that portals are associated with more favorable health outcomes for patients who use portals [30,31]. We suspect that this emerging focus on improving health outcomes through the use of portals is, in some way, connected to the end user’s satisfaction with the portal as a tool that supports their HSB. We note that this is an understudied phenomenon that is not well covered in existing research. In our paper, we focus on the patient’s satisfaction with the portal and the patient’s HSB as the outcome measure.

#### H6a: Portal Satisfaction is Positively Associated With Health-Seeking Behavior

Wixom and Todd [32] partially explained technology use intention through the influence of attitude, which was influenced by ease of use, which was, in turn, influenced by system satisfaction. Drawing from their contribution, we hypothesize that SWP will result in higher instances of portal use. This connection between satisfaction and use is also supported in the current ECT literature [21,28,32]. From the ECT literature, we note four important contributions: (1) Bhattacherjee [21] established satisfaction as an antecedent of IS continuance (repeat usage after adoption); (2) Thong et al [28] suggested end users are more likely to continue the use of an IT, if they are satisfied with that IT; (3) Oghuma et al [29] demonstrated how perceived service quality and perceived usability can impact satisfaction, which, in turn, impacts use continuance; and (4) Wixom and Todd [32] showed how system satisfaction can indirectly serve as an attitudinal influence on an end user’s intention to use a technology.

#### H6b: Portal Satisfaction is Positively Associated With Portal Use

The connection between SWP and HSB is an emerging concept. To date, there has been very little research into a direct connection between satisfaction with the portal (or health care IT of any kind) and HSB. Kim and Park [33] suggest an indirect link between health belief and the motivation to take actions toward health management. Tustin [34] explored the role that patient satisfaction plays in influencing Web-based health information seeking. Tustin suggests that patients who are dissatisfied with their health care provider are more likely to seek and trust information from sources other than their provider [34]. Although Kim and Park and Tustin attempt to explain relationships between individual factors such as health belief, level of provider satisfaction, technology, and HSB, the shared scope of HSB between these studies has been limited to Web-based health information seeking. We attempt to build upon what previous research has contributed by exploring the relationship between SWP and HSB within the context of an end user’s perceived change in health status being based on their having used a portal.

#### H7: Portal Use is Positively Associated With Health-Seeking Behavior

Some studies on understanding a patient’s motivation for connecting with Web sources, such as health websites and patient portals, are rooted in HBM. HBM suggests that a patient’s perceived health risk predicts the likelihood of that patient being more engaged in their health care. They do this by seeking more information about their health and adapt their behavior toward better health [35]. We lean on this idea that perceived severity is a determinant of perceived HSB.

#### H8a: Perceived Severity is Positively Associated With Health-Seeking Behavior

We also expect that perceived severity will be associated with increased portal use. We hypothesize that patients who perceive that they suffer from severe health conditions will demonstrate more frequent portal use.

#### H8b: Perceived Severity is Positively Associated With Portal Use

Our hypotheses are listed in Table 1. In testing these relationships, we hope to confirm existing research findings such as the work done by Hsu and Lin [36] that demonstrates the strength of social influence on a person’s motivation to use a type of IT; in their case, the focus was on the association of social influence with motivation to use blogs.

We consider the addition of the possible link between portal use and HSB to be a novel contribution that has not been heavily investigated in other research. Understanding whether this relationship exists would be informative in determining whether portal use influences HSB. This knowledge could have implications for encouraging HSB.

**Table 1:** Hypotheses tested.

Hypothesis	Hypothesis description
H_1_	Perceived social influence is positively associated with portal satisfaction.
H_2_	Habit is positively associated with portal satisfaction.
H_3_	Facilitating conditions are positively associated with portal satisfaction.
H_4_	Effort expectancy is positively associated with portal satisfaction.
H_5_	Performance expectancy is positively associated portal satisfaction.
H_6a_	Portal satisfaction is positively associated with health-seeking behavior.
H_6b_	Portal satisfaction is positively associated with portal use.
H_7_	Portal use is positively associated with health-seeking behavior.
H_8a_	Perceived severity is positively associated with health-seeking behavior.
H_8b_	Perceived severity is positively associated with portal use.

### Control Variable: Tenure

For the purposes of our research study, we define tenure as the number of days between a portal user registering for a portal account and the date on which the system report was generated to produce the dataset used in our analysis. We set a cutoff point for tenure of 60 days. This allowed us to eliminate the effect of early portal learners who could have potentially skewed the results of our study. We surmise that end users who have used the portal longer will be more satisfied, borrowing from the ECT concept of satisfaction being correlated with continued use. We also surmise that longer periods of use are more likely to be associated with perceived changes in HSB. By holding tenure as a control variable, our model allows for the testing of any direct associations between tenure and portal use as well as tenure and HSB.

## Methods

### Data Collection

Web-based survey responses were collected from a total of 1142 respondents who were active portal users within a university student health system. For the purposes of this research study, we define *active* portal users as registered portal users who logged into their portal account during the 2-year data collection period. Portal users responded to the survey via a direct link once they signed into their patient portal account. The delivery method for the survey ensured that we, at no time, were able to personally identify any patient or access specific patient details that could be linked to an individual.

We differentiate our research study from previous portal and technology use studies in that we were able to incorporate an objective measure of use. Portal use was measured as the count of portal visits for each respondent. This information was obtained from the Web logs of the patient portal system. Capturing the count of portal visits in this way facilitated the matching of portal visits to survey responses by way of masked identification numbers that were generated by the health center staff. We were able to match survey responses to portal use while simultaneously maintaining the anonymity of the portal users.

The survey items were used to obtain patient perceptions of the factors that influence their usage and their perceptions about the impact that portal usage may have on their HSB and SWP. Patients responded by providing Likert Scale-type responses (1=Strongly Disagree, 2=Disagree, 3=Neutral, 4=Agree, 5=Strongly Agree).

We also captured high-level demographic information about our survey respondents.

### Data Analysis

The mean and standard deviation for the survey responses were calculated in R Studio. Subsequently, the survey data were analyzed using partial least squares structural equation modeling (PLS-SEM) in SmartPLS. PLS-SEM is an appropriate technique for testing relationships within the proposed theoretical model because of the use of latent variables [37]. Given that all of the indicators in our model are reflective, we evaluated the reliability of each construct through the use of Cronbach alpha and composite reliability. To assess convergent validity, we evaluated the average variance extracted (AVE). Discriminant validity was assessed to determine significant interconstruct differences [38]. We evaluated the heterotrait-monotrait ratio of correlations in an effort to determine if discriminant validity exists between the reflective constructs in our proposed model.

## Results

### Demographic Data

Of the 1142 survey respondents, 705 (61.73%) were female and 437 (38.27%) were male (Table 2).

Ethnicity was self-reported as African American, Native American, Asian or Pacific islander, white, any two or more races, international or unspecified. People that identified as white represented the highest number of respondents, 52.36% (598/1142). People that identified as Native American were among the fewest survey respondents. Only 3 people identified as Native American. Academic standing was recorded as freshman, sophomore, junior, senior, fifth year, or graduate students. Freshmen comprised the largest number of survey respondents, 47.46% (542/1142) and fifth year students comprised the smallest number of respondents, 0.79% (9/1142).

**Table 2:** Survey respondent demographic data (n=1142).

Characteristics		Value, n
**Gender**		
	Male	437
	Female	705
**Ethnicity**		
	African American	172
	Native American	3
	Any two or more races	82
	Asian or Pacific islander	93
	White	598
	International	68
	Not specified	126
**Academic standing**		
	Freshman	542
	Sophomore	206
	Junior	184
	Senior	81
	Fifth year	9
	Graduate	108
	Early college	12

### Reliability

Mean response scores and reliability analysis are presented in Table 3.

Social influence, effort expectancy, performance expectancy, HSB, and SWP demonstrated Cronbach alphas that were greater than .80. Habit and facilitating conditions both had weaker Cronbach alpha of .726 and .710, respectively. Perceived severity had the lowest Cronbach alpha of .611, and it actually fell below the widely accepted threshold of .70 [39]. Owing to the exploratory nature of our research study, we also reviewed acceptable thresholds for composite reliability. We noted that the composite reliability for perceived severity exceeded 0.60, which has been viewed as acceptable in an exploratory research context [40]. We therefore retained perceived severity in our model analysis.

We found that all of the values for AVE in our analysis (Table 3) were higher than the Fornell and Locker [41] suggested threshold of 0.50 for AVE. This leads us to conclude that there is sufficient convergent validity for each of our model constructs.

**Table 3:** Mean response scores and reliability analysis.

Theoretical construct and survey item			Value, mean (SD)		Outer loadings		Cronbach alpha		Composite reliability		Average variance extracted
**S^a^**							.846		0.907		0.766
	S1—People who care for me want me to use the portal.	3.91 (0.872)		0.896							
	S2—People who influence me want me to use the portal.	3.76 (0.871)		0.916							
	S3—My nurse or physician has encouraged me to use the portal.	3.40 (0.910)		0.811							
**H^b^**							.726		0.879		0.784
	H1—Portal usage has become a habit for me.	3.19 (0.916)		0.896							
	H2—I must use the portal on a regular basis to improve my health.	3.21 (0.937)		0.875							
**FC^c^**							.710		0.873		0.775
	FC1—I know how to access the portal.	4.26 (0.821)		0.887							
	FC2—The portal works with other technology that I use.	4.16 (0.784)		0.874							
**EE^d^**							.894		0.950		0.904
	EE1—Learning to use the portal was easy for me.	4.02 (0.834)		0.948							
	EE2—The portal was easy to navigate and use.	4.04 (0.807)		0.953							
**PE^e^**							.870		0.939		0.885
	PE1—Using the portal will support the care I receive.	3.91 (0.769)		0.943							
	PE2—Using the portal allows me to be more involved in my own care.	3.95 (0.769)		0.939							
**PS^f^**							.611		0.835		0.717
	PS1—I believe I am vulnerable to illnesses.	2.60 (1.100)		0.886							
	PS2—I believe that my current health conditions are serious.	2.07 (1.039)		0.806							
**HSB^g^**							.910		0.957		0.917
	HSB1—Portal usage has influenced me to adopt healthier behaviors.	3.18 (0.840)		0.957							
	HSB2—Portal usage has influenced me to exercise more.	3.06 (0.861)		0.959							
**SWP^h^**							.824		0.919		0.850
	SWP1—I am satisfied with the patient portal.	3.92 (0.734)		0.916							
	SWP2—I would recommend using the portal to my friends and family.	3.73 (0.795)		0.928							

^a^S: social influence.

^b^H: habit.

^c^FC: facilitating conditions.

^d^EE: effort expectancy.

^e^PE: performance expectancy.

^f^PS: perceived severity.

^g^HSB: health-seeking behavior.

^h^SWP: portal satisfaction.

### Discriminant Validity

Discriminant validity analysis is presented in Table 4.

None of the correlation values in Table 4 exceeds the widely accepted threshold of 0.90 for heterotrait-monotrait values [42,43]. Owing to this observation, we assert that the independent variables in our model demonstrate sufficient discriminant validity.

**Table 4:** Discriminant validity.

Model constructs	Effort expectancy	Facilitating conditions	Habit	Health-seeking behavior	Perceived severity	Performance expectancy	Satisfaction with portal	Social influence	Tenure
Effort expectancy	N/A^a^	N/A	N/A	N/A	N/A	N/A	N/A	N/A	N/A
Facilitating conditions	0.821	N/A	N/A	N/A	N/A	N/A	N/A	N/A	N/A
Habit	0.419	0.443	N/A	N/A	N/A	N/A	N/A	N/A	N/A
Health-seeking behavior	0.362	0.305	0.712	N/A	N/A	N/A	N/A	N/A	N/A
Perceived severity	0.049	0.085	0.239	0.202	N/A	N/A	N/A	N/A	N/A
Performance expectancy	0.685	0.672	0.627	0.535	0.155	N/A	N/A	N/A	N/A
Satisfaction with portal	0.775	0.717	0.665	0.592	0.094	0.857	N/A	N/A	N/A
Social influence	0.402	0.452	0.793	0.564	0.167	0.646	0.67	N/A	N/A
Tenure	0.020	0.024	0.090	0.095	0.156	0.018	0.017	0.106	N/A
Use	0.035	0.021	0.141	0.015	0.106	0.019	0.058	0.06	0.568

^a^Not applicable.

### Path Analysis

To test our research hypotheses, we used the results from PLS-SEM. In our initial tests, we conducted the model analysis with a bootstrapping sample of 500. In an effort to assess the robustness of our findings, we subsequently ran the model with a bootstrapping sample of 1000. The overall results of our analysis did not materially change between the two model runs with different bootstrapping sample sizes. The significance of established associations remained consistent between the two iterations of our analysis.

**Figure 2:**
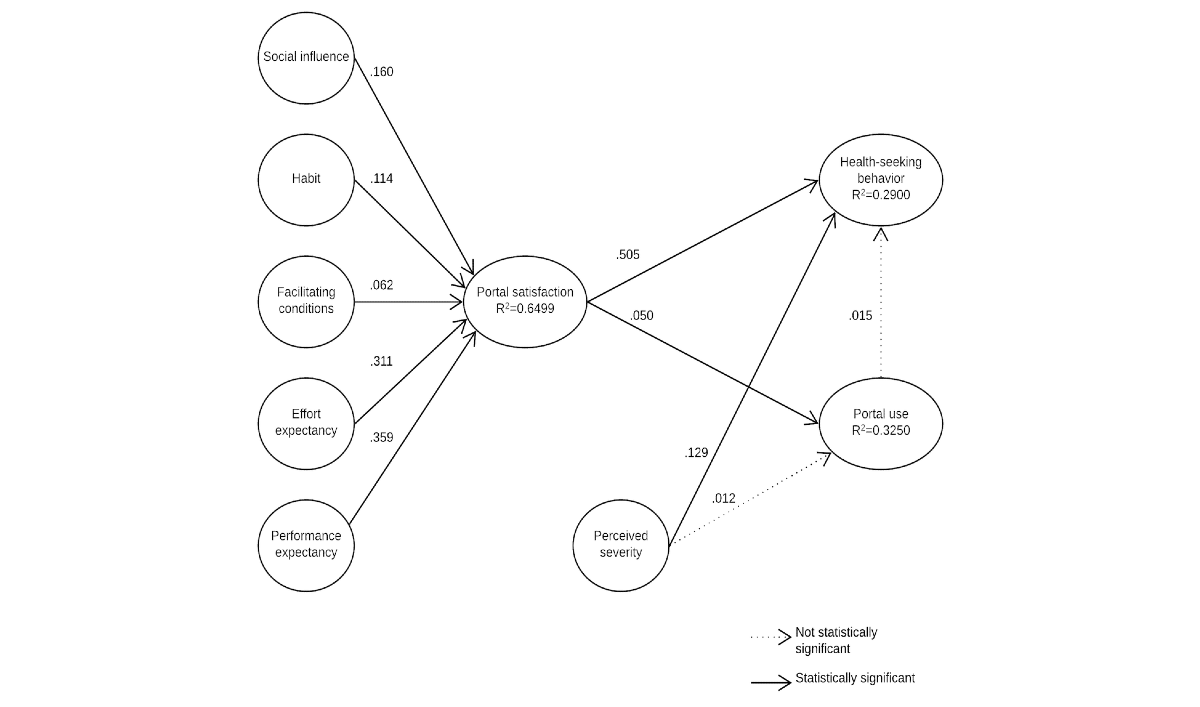
Empirical model with path coefficients.

Our model explains 29% of the variance observed in HSB (R^2^=0.2900) and 65% of the variance observed in SWP (R^2^=0.6499). The results from our PLS-SEM (Figure 2) suggest significant associations among social influence, habit, facilitating conditions, effort expectancy, performance expectancy, and SWP. Perceived severity is significantly associated with HSB, but it is not significantly associated with portal use. Portal use, contrary to our initial supposition, is not significantly associated with HSB.

We found support for 8 out of 10 of our hypotheses (Table 5). The strongest association observed in our model appears to be between SWP and HSB, demonstrated by a path coefficient of .505 (*P*<.001). We also found that tenure is significantly associated with both HSB and portal use. As tenure was treated as a control variable, we did not draw any major conclusions about level of significance.

**Table 5:** Path analysis results: full model.

Dependent and independent variables		Path coefficient (beta)	Standard error	*t* statistic (*df*=499)^a^	Hypothesis	Supported (yes/no)	R^2^	
**Portal satisfaction**								0.6499
	Social influence	.160	0.025	6.145^b^	H_1_	Yes		
	Habit	.114	0.023	4.89^b^	H_2_	Yes		
	Facilitating conditions	.062	0.026	2.401^c^	H_3_	Yes		
	Effort expectancy	.311	0.028	11.149^b^	H_4_	Yes		
	Performance expectancy	.359	0.031	11.588^b^	H_5_	Yes		
**Health-seeking behavior**								0.2900
	Portal satisfaction	.505	0.026	19.705^b^	H_6a_	Yes		
	Use	.015	0.028	.513	H_7_	No		
	Perceived severity	.129	0.028	4.675^b^	H_8a_	Yes		
	Tenure	−.116	0.030	3.925^b^	Control	N/A^d^		
**Portal use**								0.3250
	Portal satisfaction	.050	0.025	2.031^c^	H_6b_	Yes		
	Perceived severity	.012	0.025	.488	H_8b_	No		
	Tenure	.566	0.039	14.409^b^	Control	N/A		

^a^2-tailed *t* test.

^b^*P*<.1.

^c^*P*<.05.

^d^Not applicable.

## Discussion

### Principal Findings

#### RQ1. What Factors Are Associated With a Patient’s Portal Satisfaction?

We find that SWP is driven most strongly by performance expectancy. If we rely on Venkatesh et al’s [14] definition of performance expectancy, wherein performance expectancy is understood to represent the degree to which users expect systems to help them attain a specific performance, we note that portal user satisfaction appears to be driven mostly by what portal users think they will get out of using the system. Our research supports social influence, habit, facilitating conditions, and effort expectancy as factors that are associated with SWP.

#### RQ2. Does Patient Portal Satisfaction Influence Health-Seeking Behavior?

Our analysis suggests that HSB is significantly associated with SWP. The association between HSB and SWP was the highest association that our model demonstrated between any of the model constructs with a path coefficient of .505.

#### RQ3. Does Patient Portal Use Influence Health-Seeking Behavior?

Our empirical analysis does not support a relationship between portal use and HSB. We had expected to find a positive influence of portal usage on HSB. Intuitively, as patients more frequently use portals, one would expect to see a strong, positive correlation between this portal use and HSB. Our analysis, however, demonstrates no significant link between portal use and HSB.

#### RQ4. Does Patient Portal Satisfaction Influence Portal Use?

Patient SWP does appear to influence portal use. We observed a significant positive correlation between SWP and portal use. Although this correlation was not as strong as we had anticipated (beta=.050), the correlation between the two constructs was statistically significant (*P*=.04). This result does add to the existing ECT literature in that it supports the idea that use (or continued use in ECT parlance) can be influenced by end user satisfaction. Our study supports the work of Thong et al [28] in that the relationship between satisfaction and continued use suggests a need for IT developers to focus on developing easy-to-use technology features as a method for ensuring satisfaction and, thereby, driving continued technology use.

#### RQ5. Does Perceived Severity of Health Condition Influence Health-Seeking Behavior?

Perceived severity of health condition has a weak, positive association with HSB. Intuitively, one would expect to find that the more severe a patient’s condition, the more frequently the patient would exhibit HSB. Although we found only a weak, positive association between perceived severity and HSB, the association between the two is statistically significant. This association would have been missed had we eliminated it from our study by solely relying on the Cronbach alpha of .611 (Table 3).

#### RQ6. Does Perceived Severity of Health Condition Influence Portal Use?

Perceived severity of health condition does not have a significant association with portal use. This finding was counterintuitive in that we expected perceived severity to play a strong role in driving portal use. We expected to find that survey respondents who perceived themselves to have more severe health conditions would report higher levels of portal use. This, however, was not the case.

### Limitations

The authors acknowledge geographic limitations to this study. Data were collected and analyzed from a single academic institution. It is possible that there are unique characteristics of this college student population that may prevent the generalizability of our findings to other populations of portal users. These characteristics might include age, education level, technology literacy, and easy access to technology. Future research should include data from the general population for the purposes of comparing, contrasting, and possibly strengthening the generalizability of the findings. The data used in this study were collected using a snapshot of observations within a 2-year time frame. Hence, the study suffers from the general limitations of using snapshot data [44]. Future longitudinal studies can address this limitation and provide greater insight into portal user perceptions over time. Longitudinal studies generally provide more statistically powerful tests [45].

### Conclusions

Our research benefited from a sample size of 1142 portal user survey responses. Also, unlike many previous studies, our research benefited from objective data on actual portal use. We tested the overall reliability of the responses, and we found support for existing theories of how concepts from TAMs can be used to explain associations between those widely accepted concepts, and SWP might serve as a proxy for technology satisfaction in future research. We also found utility in these widely accepted technology acceptance constructs as a means of explaining some aspects of HSB.

Our findings, interestingly, showed a minor significant role for perceived severity as it relates to perceived HSB. Although the effects were small, they were significant. This relationship may spark future research interests into the role that perceived severity may play in understanding perceived HSB.

We also determined that there was a link between SWP and HSB. This link may also spark future research prompted by the desire to better understand whether SWP is likely to be a determinant of HSB or health information seeking.

## Abbreviations

AVE: average variance extracted
ECT: Expectation-Confirmation Theory
EHR: electronic health record
HBM: Health Belief Model
HSB: health-seeking behavior
IS: information system
IT: information technology
PLS-SEM: partial least squares structural equation modeling
RQ: research question
SWP: portal satisfaction
TAM: Technology Acceptance Model
UTAUT2: Unified Theory of the Acceptance and Use of Technology 2
